# The Impact of Systemic Lupus Erythematosus-Related Respiratory Manifestations on the Quality of Life and Psychological Health of Patients During the COVID-19 Pandemic

**DOI:** 10.7759/cureus.38282

**Published:** 2023-04-29

**Authors:** Ibtissam El Harch, Naoual Oubelkacem, Mohammed Omari, Soumaya Benmaamar, Bineta Jho Diagne, Nada Otmani, Nabil Tachfouti, Rhizlane Berrady, Samira El Fakir

**Affiliations:** 1 Laboratory of Epidemiology, Clinical Research and Community Health, Sidi Mohamed Ben Abdellah University, Faculty of Medicine and Pharmacy, Fes, MAR; 2 Internal Medicine Department, Centre Hospitalier Universitaire Hassan II, Fes, MAR

**Keywords:** quality-of-life, systemic lupus erythematosus related respiratory manifestations, depression, anxiety, covid 19, systemic lupus erythematosus disease

## Abstract

Introduction

Respiratory manifestations are common among patients with Systemic Lupus Erythematosus (SLE) and can present as chest pain, dyspnea, and cough and are often accompanied by fever. These symptoms can resemble those of COVID-19, which may cause increased anxiety in SLE patients. Therefore, the aim of this study is to investigate the impact of SLE-related respiratory manifestations on anxiety, depression, and quality of life among SLE patients during the COVID-19 pandemic.

Patients and methods

The study involved SLE patients and was conducted in the year 2020, after the start of the pandemic in Morocco, using a cross-sectional design. Anxiety and depression were evaluated using the Hospital Anxiety and Depression Scale (HADS), while the quality of life was assessed using the Short Form-12 Health Survey (SF12). Statistical analysis was performed using R software (R Foundation, Vienna, Austria).

Results

A total of 102 SLE patients, with an average age of 41.6 ± 13.7 years, participated in the study, of whom 92.2% were female. Respiratory manifestations were reported by 20.6% of the patients, and there were no significant differences observed in the general characteristics of the study population between the two groups with and without SLE-related respiratory manifestations.

The study found that the prevalence of anxiety and depression was significantly higher in patients with SLE-related respiratory manifestations (50% Vs. 76,2% and 50% Vs. 85,7% successively). These patients also reported significantly more impairment in their physical quality of life (31.8 ± 8.9 Vs. 38.5 ± 10.9). This was observed across three domains of the SF12 survey, including physical functioning (34.4 ± 11.4 Vs. 39.9 ± 11.7), bodily pain (26.9 ± 11.2 Vs. 36.1 ± 14.3), and general health (28.6 ± 10.7 Vs. 35.2 ± 12.3). Although the association between mental quality of life and respiratory manifestations did not reach statistical significance (33.5 ± 12.5 Vs. 39.1 ± 11.5), there was a trend toward poorer mental quality of life in patients with SLE-related respiratory manifestations. Moreover, two domains of mental quality of life were significantly more affected in these patients, namely “social functioning” (30.6 ± 11.3 Vs. 38.7 ± 12.4) and “role-emotional” (26.8 ± 11.6 Vs. 33.8 ± 10.8).

Conclusion

During the COVID-19 pandemic, the presence of SLE-related respiratory manifestations appeared to be associated with a more negative impact on the psychological health and quality of life of SLE patients.

## Introduction

Systemic lupus erythematosus (SLE) is a complex chronic autoimmune disease [1] that mainly affects young women [2]. The disease is characterized by a broad spectrum of clinical manifestations ranging from skin disease to multiple organ failure [3,4].

Several studies have indicated that a considerable proportion (20%-90%) of patients with SLE experience respiratory issues [5,6]. More recent research suggests that this figure is between 50% and 70% [7]. The most common respiratory symptoms reported are pleuritic or musculoskeletal chest pain, dyspnoea, cough, and hemoptysis, often accompanied by fever [8].

COVID-19, which first appeared in Wuhan, China, in December 2019 [9,10], mainly affects the lungs and is caused by SARS-CoV-2 [11]. The major symptoms are cough and dyspnoea associated with fever [12]. In Morocco, the first case of COVID-19 was reported on March 2, 2020 [13]. The World Health Organization declared COVID-19 as a pandemic [14] on March 11, 2020. Within 6 months, the virus infected more than 10 million people worldwide and caused more than 500,000 deaths [15]. This pandemic has caused anxiety and depression among people with chronic diseases, who have been particularly susceptible to the virus [16,17], including those with lupus [18].

The rapid global spread of COVID-19 and the unknown nature of the disease have had a major impact on people’s mental health, causing anxiety, depression, and reduced quality of life [19-21], particularly among those with SLE. [22].

A recent study compared the mental health status of SLE patients during the COVID-19 lockdown period to that of a group evaluated before the pandemic. The results showed a statistically significant decline in mental health among the lockdown patient group, with increased vulnerability to stress, depression, anxiety, phobic anxiety, and interpersonal sensitivity [23].

COVID-19 and SLE have notable similarities in their respiratory symptoms. As a result, the pandemic's impact on patients with SLE-related respiratory manifestations could be amplified. Therefore, the primary aim of this study is to examine the effect of the presence of SLE-related respiratory symptoms on anxiety, depression, and quality of life among SLE patients during the pandemic.

## Materials and methods

Study design

This is a cross-sectional study conducted during the COVID-19 pandemic period (December 2020 to December 2021) in the Internal Medicine Department of Hassan II University Hospital Center in Fes, Morocco.

Study population

This study included patients aged 18 years and above, diagnosed with SLE according to American College of Rheumatology (ACR) criteria [24], and treated in the Internal Medicine Department of Hassan II University Hospital Center in Fez. Patients with documented intellectual disability, major psychopathology or neurocognitive disorders were excluded. Patients were considered to have SLE-related respiratory manifestations if they had clinically, radiologically, or histologically confirmed respiratory involvement.

Data collection

After obtaining approval from the hospital-university ethics committee (approval number 26/18), eligible patients were invited to participate. Those who agreed provided written consent and answered a questionnaire that included inquiries about their socio-demographic characteristics, such as age, gender, marital status, education level, employment status, place of residence, as well as their medical history. Information on the duration of the disease and the different manifestations of lupus was obtained by interviewing the patients or by reviewing their medical records. Anxiety and depression were measured using the Hospital Anxiety and Depression Scale (HADS), while the quality of life was measured using the Short Form 12 scale (SF12).

Hospital Anxiety and Depression Scale (HADS)

The HADS was developed by Zigmond and Snaith in 1983. It is a self-report scale that identifies anxiety and depressive disorders. It contains 14 items, seven related to anxiety (total A) and seven others related to depression (total D). For each item, the response is scored from 0 to 3 on a scale depending on the intensity of the symptom during the past week. The possible scores extend for each subscale from 0 to 21. The highest scores correspond to the presence of more severe symptoms. For each subscale (anxiety and depression), cutoff values were determined: a score between 0 and 7 is considered normal, while a score of 8 or higher indicates a significant disorder [25,26].

Short Form 12 scale

The SF12 scale is constructed from the SF36 scale, developed and analyzed by Ware and Sherbourne [27]. Its validation was carried out with 9000 people in nine European countries [28]. The SF12 has been validated in Morocco for assessing the state of health of the Moroccan population [29]. Responses to the SF12 are used to establish two scores, one for physical quality of life and another for mental quality of life. Each score is divided into four domains:

Physical health (PCS): 1) Physical functioning (PF), 2) Role-physical (RP), 3) Bodily Pain (BP), 4) General health (GH)

Mental health (MCS): 1) Vitality (VT), 2) Social functioning (SF), 3) Role-emotional (RE), 4) Mental health (MH).

The scores for each subscale range from 0 to 100, with higher scores reflecting better quality of life.

Statistical analysis

Descriptive statistics were used to summarize the characteristics of patients, including age, sex, marital status, education level, occupational status, housing, medical history, duration of illness, SLE manifestations, anxiety, depression, and quality of life. Frequency was used to report qualitative variables, while mean and standard deviation were used to represent quantitative variables.

A comparison of the general characteristics was conducted between the two groups (those with and without SLE-related respiratory manifestations), using the Student's t-test to compare means and the Chi-square or Fisher's exact test to compare percentages.

Logistic regression was used to explore the association between the presence of SLE-related respiratory manifestations and the likelihood of experiencing anxiety and depression. The findings were presented as odds ratios (OR) and their 95% confidence intervals (CI95%).

After verifying the normality of PCS and MCS, a simple linear regression was utilized to investigate the correlation between the presence of SLE-related respiratory manifestations and quality of life. The outcomes were displayed as regression coefficients (β) and their CI95%. R software (R Foundation, Vienna, Austria) was used to conduct the statistical analysis.

## Results

A total of 102 SLE patients participated in the study, the majority of which (92.2%) were women. The average age of the patients was 41.6 ± 13.7 years, 50.0% had a low level of education, 59.8% were married, 68.3% lived in urban areas and only 8.8% lived alone. For the medical histories, 87.3% had high blood pressure, 9.8% had diabetes, and 12.7% had another autoimmune disease. In patients with organ involvement, dermatological involvement was seen in 70.6%, rheumatological involvement in 64.7%, renal involvement in 50%, respiratory involvement in 20.6%, and vascular involvement in 11.8% of cases. The average duration of disease was 6.8 ± 5.5 years (Table 1).

**Table 1:** General characteristics of the total study population (N = 102)

		N	%
Age (m ± sd years)		41.6 ± 13.7	
Sex	Women	94	92.2%
	Men	8	7.8%
Level of education (n = 100)	Low level of education	50	50%
	High level of education	50	50%
Employment status	Unemployed	74	72.5%
	Employed	28	27.5%
Marital status	Not married	41	40.2%
	Married	61	59.8%
Habitat (N =101)	Urban	69	68.3%
	Rural	32	31.7%
Housing	Only	9	8.8%
	With husband OR with family	93	91.2%
Arterial hypertension		13	12.7%
Diabetes		10	9.8%
Cadiopathy		10	9.8%
Nephropathy		8	7.8%
Neoplasia		1	1%
Other autoimmune diseases		13	12.7%
Dermatological manifestations		72	70.6%
Rheumatological manifestations		66	64.7%
Renal manifestations		51	50.0%
Vascular manifestations		12	11.8%
Respiratory manifestations		21	20.6%
Anti-DNA antibodies (N = 83)		39	47.0%
Anti-nuclear antibodies (N = 84)		17	20.2%
Duration of disease in years (m ± sd)		6.8 ± 5.5	

The comparison of general characteristics between the study population with and without SLE-related respiratory manifestations was shown. However, no significant differences were found between the two groups (see Table 2).

**Table 2:** Comparison of baseline characteristics between the two groups with and without SLE-related respiratory manifestations SLE: Systemic Lupus Erythematosus

		SLE-related respiratory manifestations		p-value
		No N = 81 (79.4%)	Yes N = 21 (20.6%)	
Age (m ± sd) years		40.6 ± 13.3	45.8 ± 14.9	0.123
Gender	Women	76 (93.8%)	18 (85.7%)	0.356
	Men	05 (6.2%)	03 (14.3%)	
Level of study (N = 100)	Low level of study	40 (50.6%)	10 (47.6%)	1.000
	High level of study	39 (49.4%)	11 (52.4%)	
Profession	Unemployed	56 (69.1%)	18 (85.7%)	0.173
	Employed	25 (30.9%)	03 (14.3%)	
Marital status	Not married	33 (40.7%)	08 (38.1%)	1.000
	Married	48 (59.3%)	13 (61.9%)	
Habitat (N =101)	Urban	56 (70.0%)	13 (61.9%)	0.599
	Rural	24 (30.0%)	08 (38.1%)	
Housing	Only	8 (9.9%)	1 (4.8%)	0.681
	With husband OR with family	73 (90.1%)	20 (95.2%)	
Arterial hypertension	No	70 (86.4%)	19 (90.5%)	1.000
	Yes	11 (13.6%)	02 (9.5%)	
Diabetes	No	74 (91.4%)	18 (85.7%)	0.426
	Yes	07 (8.6%)	03 (14.3%)	
Cardiopathy	No	73 (90.1%)	19 (90.5%)	1.000
	Yes	08 (9.9%)	02 (9.5%)	
Nephropathy	No	74 (91.4%)	20 (95.2%)	1.000
	Yes	07 (8.6%)	01 (4.8%)	
Neoplasia	No	80 (98.8%)	21 (100.0%)	1.000
	Yes	01 (1.2%)	00 (0.0%)	
Other autoimmune diseases	No	72 (88.9%)	17 (81.0%)	0.461
	Yes	09 (11.1%)	04 (19.0%)	
Dermatological manifestations	No	22 (27.2%)	8 (38.1%)	0.421
	Yes	59 (72.8%)	13 (61.9%)	
Rheumatological manifestations	No	29 (35.8%)	7 (33.3%)	1.000
	Yes	52 (64.2%)	14 (66.7%)	
Renal manifestations	No	42 (51.9%)	9 (42.9%)	0.625
	Yes	39 (48.1%)	12 (57.1%)	
Vascular manifestations	No	74 (91.4%)	16 (76.2%)	0.068
	Yes	7 (8.6%)	5 (23.8%)	
Anti-DNA antibodies	Negatives	33 (50.8%)	11 (61.1%)	0.595
	Positives	32 (49.2%)	7 (38.9%)	
Anti-nuclear antibodies	Negatives	50 (76.9%)	17 (89.5%)	0.336
	Positives	15 (23.1%)	2 (10.5%)	
Duration of disease (m ± sd years)		6.5 ± 5.1	7.71 ± 6.9	0.480

Table 3 shows that 76.2% of patients with SLE-related respiratory manifestations had anxiety, while 85.7% had depression. Statistical analysis revealed that the risk of anxiety and depression was significantly higher in patients with SLE-related respiratory manifestations compared to those without, with an OR of 3.2 (95% CI = 1.1 - 9.6) and 6.0 (95% CI = 1.6 - 21.9), respectively.

**Table 3:** HADS and SF12 scales description and their association with SLE-related Respiratory manifestations PCS: Physical health score; PF: Physical functioning; RP: Role-physical; BP: Bodily pain; GH: General health; MCS: Mental health score; VT: Vitality; SF: Social functioning; RE: Role-emotional; MH: Mental health; HADS: Hospital Anxiety and Depression Scale; SF12: Short Form-12; SLE: Systemic Lupus Erythematosus *(m ± sd); °Crude OR and CI 95%

		Descriptive analysis		Univariate analysis	
		SLE-related Respiratory manifestations		Crude ꞵ (CI 95%)	p-value
		No	Yes		
Anxiety		40 (50.0%)	16 (76.2%)	3.2 (1.1 - 9.6)°	0.037
Depression		40 (50.0%)	18 (85.7%)	6.0 (1.6 – 21.9)°	0.007
PCS		38.5 ± 10.9*	31.8 ± 8.9*	6.7 (1.5 – 11.9)	0.011
	PF	39.9 ± 11.7*	34.4 ± 11.4*	5.5 (-0.1 – 11.2)	0.054
	RP	37.6 ± 9.7*	31.1 ± 9.3*	6.5 (1.8 – 11.1)	0.007
	BP	36.1 ± 14.3*	26.9 ± 11.2*	9.2 (2.5 – 15.8)	0.007
	GH	35.2 ± 12.3*	28.6 ± 10.7*	6.6 (0.8 – 12.4)	0.027
MCS		39.1 ± 11.5*	33.5 ± 12.5*	5.6 (-0.1 – 11.2)	0.054
	VT	44.0 ± 10.7*	40.6 ± 11.1*	3.5 (-1.8 – 8.7)	0.194
	SF	38.7 ± 12.4*	30.6 ± 11.3*	8.1 (2.2 – 14.1)	0.008
	RE	33.8 ± 10.8*	26.8 ± 11.6*	6.9 (1.7 – 12.3)	0.011
	MH	40.0 ± 13.6*	34.6 ± 13.3*	5.4 (-1.2 – 11.9)	0.109

Physical quality of life was impaired in patients with SLE-related respiratory manifestations compared to those without these manifestations (31.8 ± 8.9 vs. 38.5 ± 10.9). This impairment was statistically significant (β= 6.7; CI95% = 1.5 - 11.9). The same finding was observed for three of the four domains of this type of quality of life, namely PR (β = 6.5; CI95% = 1.8 - 11.1), BP (β = 9.2; CI95% = 2.5 - 15.8), and GH (β = 6.6; CI95% = 0.8 - 12.4) (Table 3).

Moreover, mental quality of life was also impaired in patients with SLE-related respiratory manifestations compared to those without (β= 33.5 ± 12.5 vs. 39.1 ± 11.5). This difference was borderline significant (β= 5.6; 95% CI =-0.1 - 11.2 and p =0.054). Among the four domains of mental quality of life, two were significantly more impaired in patients with SLE-related respiratory manifestations, namely SF and RE (β= 8.1; CI95% = 2.2 - 14.1 and β= 6.9; CI95% = 1.7 - 12.3, respectively) (Table 3).

## Discussion

The main objective of this study was to assess the impact of SLE-related respiratory manifestations on patients’ quality of life and psychological health during the COVID-19 pandemic. Our findings indicate that patients with SLE-related respiratory manifestations had a significantly poorer physical and mental quality of life and were more anxious and depressed compared to those without such manifestations. This negative impact can be explained by the patients’ fear of having an underlying COVID-19 infection, due to the high similarity between the respiratory manifestations of SLE and those of COVID-19 [8,30]. Due to their underlying health conditions, this vulnerable population is aware that they are at a higher risk of COVID-19 infection, including a higher risk of hospitalization, longer hospital stays, and even death from COVID-19, as demonstrated by several studies. For instance, an Italian study reported a prevalence of COVID-19 of 3.4% in SLE patients compared to 0.33% in the general population [31]. Another study conducted in France based on the data of all patients hospitalized during the first 6 months of the pandemic found that among 1,411 SLE patients hospitalized with COVID-19, 17% required intensive care unit hospitalization and 9.5% died [32]. In addition, a study of 2,140 SLE patients who contracted COVID-19 confirmed that these patients had a higher risk of hospitalization, admission to intensive care units, mechanical ventilation, strokes, and sepsis compared to the general population [18]. The age- and sex-adjusted hospitalization rate per 1,000 person-years was 6.16 (95% CI: 3.76-10.08) in SLE patients and 2.54 (95% CI: 1.55-4.16) in the matched group from the general population [33].

Although the prevalence of anxiety and depression was significantly higher in patients with SLE-related respiratory manifestations, this study showed that patients without any type of SLE-related respiratory manifestations still had a prevalence rate of 50% of anxiety and depression, as well as physical and mental quality of life values below 50. These findings suggest that the presence of respiratory manifestations linked only to SLE exacerbates the deterioration of the quality of life and increases the prevalence of anxiety and depression, which were already present in SLE patients during the pandemic. This is confirmed in a study conducted on people with SLE living in the Northeastern United States which showed that the pandemic had caused them severe physical and emotional stress through an increase in anxiety and depression, as well as stress caused by the fear and uncertainty of being chronically immunocompromised [22]. Similarly, a study conducted in the United States, which compared the prevalence of depression in SLE patients before and after the pandemic, found that depression, which was already present in these patients, increased more than threefold during the pandemic, from 8.5% before to 27.8% during COVID-19 [20].

This study has some benefits. Firstly, it is the first study to examine the impact of SLE-related respiratory manifestations on the psychological health and quality of life of patients during the pandemic. Secondly, it includes patients who are treated at a University Hospital Center which receives patients from across the Fes-Meknes region. However, the study is limited by the fact that it is a single-center study, based on a small sample size and relies on self-reported questionnaires to assess quality of life, anxiety, and depression.

## Conclusions

In conclusion, this study has reaffirmed that patients with SLE face significant deterioration in their quality of life and psychological health. However, the additional burden of the COVID-19 pandemic on SLE-respiratory manifestations patients has exacerbated these challenges. As such, it is essential for healthcare providers to adopt a holistic approach to caring for these patients, whether during or outside pandemics, by addressing their physical, psychological, and emotional needs.
